# Collective Response of Zebrafish Shoals to a Free-Swimming Robotic Fish

**DOI:** 10.1371/journal.pone.0076123

**Published:** 2013-10-16

**Authors:** Sachit Butail, Tiziana Bartolini, Maurizio Porfiri

**Affiliations:** Department of Mechanical and Aerospace Engineering, Polytechnic Institute of New York University, Brooklyn, New York, United States of America; Universitat de Barcelona, Spain

## Abstract

In this work, we explore the feasibility of regulating the collective behavior of zebrafish with a free-swimming robotic fish. The visual cues elicited by the robot are inspired by salient features of attraction in zebrafish and include enhanced coloration, aspect ratio of a fertile female, and carangiform/subcarangiform locomotion. The robot is autonomously controlled with an online multi-target tracking system and swims in circular trajectories in the presence of groups of zebrafish. We investigate the collective response of zebrafish to changes in robot speed, achieved by varying its tail-beat frequency. Our results show that the speed of the robot is a determinant of group cohesion, quantified through zebrafish nearest-neighbor distance, which increases with the speed of the robot until it reaches 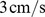. We also find that the presence of the robot causes a significant decrease in the group speed, which is not accompanied by an increase in the freezing response of the subjects. Findings of this study are expected to inform the design of experimental protocols that leverage the use of robots to study the zebrafish animal model.

## Introduction

The use of engineered stimuli to study animal behavior is both inspired by and aids in the understanding of animal perception. In this context, animated images [1], [2], virtual projections [3], and robots [4], [5] have been recently integrated into the design of experimental protocols to conduct hypothesis-driven studies of animal behavior. Among these, robots are expected to provide the largest degree of control through the exploitation of other sensory modalities beyond vision [6] and through their three-dimensional presence and mobility in the environment [7]. These features allow for the synthesis of a dynamic, yet controllable, stimulus that can elicit a meaningful response in the experimental subjects[4], [8].

This interdisciplinary research area at the interface of ethology and robotics is often referred to as ''ethorobotics'' [6], [9]. Existing robot designs for ethorobotics research can be broadly classified in two categories based on their mobility. Those that mimic animal body movements while being anchored to a single location [6], [10]–[19] and those that cover spatial ground without any appreciable body movement [7], [8], [20]–[23]. Anchored robots that mimic body movements have been used to study multi-sensory alarm signals in squirrels [6], male courtship response to female behavior in satin bowerbirds [10], social information transfer in three different bird species [19], and fish response to biomimetic locomotion [11], [14]. Spatial control of robots has been particularly effective in the study of group response. For example, mobile robots releasing pheromones in the environment were used to explore shelter seeking in cockroaches [7], robotic replicas moving on a guide line were used to study quorum decision making in sticklebacks [8], and a robotic sheepdog was used to control a flock of ducks [20]. In freshwater fish species that use vision as a primary sensory modality, both these form of movements have been used individually to modulate animal behavior [12]–[15], [17], [22], [24].

In this work, we integrate these two forms of movements in a robotic fish that propels itself through a carangiform/subcarangiform body undulation to freely swim in a test tank. The free-swimming capability allows the robot to actively interact with live subjects in different areas of the experimental tank, while the body undulations are expected to enhance its degree of biomimicry. Moreover, body undulations can elicit salient flow cues [11], which have been shown to be determining factors in the spatial arrangements of fish schools [25], [26]. We utilize such robotic fish to investigate the collective response of groups of zebrafish.

Zebrafish (*Danio rerio*) are rapidly emerging as an experimental species for the investigation of functional and dysfunctional biological processes, due to the sequencing of their genome, their high reproduction rate, short intergeneration time, prominent shoaling tendency, and elevated stocking density compared to laboratory mammals [27]–[32]. For example, zebrafish are extensively used to study the effects of drugs of abuse and ethanol administration [33]–[36]. Furthermore, with the aim of generating high-throughput behavioral data [37], considerable research is being performed to integrate animated images of conspecifics, heterospecifics [38], [39], and predators [2] in experimental paradigms. In the context of ethorobotics, we have recently proposed a series of dichotomous preference tests to study zebrafish response to an anchored robotic fish whose design is inspired by salient features of attraction in zebrafish. Specifically, in [12], it is shown that zebrafish responds differentially to variation in aspect ratio and color in the robotic fish; in [13], it is demonstrated that zebrafish shoals prefer such a robotic fish to an empty compartment; in [14], it is demonstrated that an interactive robot, whose tail-beat frequency responds to fish position, is able to induce preference among single organisms; and in [17], it is shown that the robotic fish is able to simultaneously attract shoals of zebrafish while repelling shoals of mosquitofish that would otherwise display aggressive behavior. Finally, in [16], the robotic fish is utilized as a tool to analyze the effect of ethanol administration on zebrafish behavior.

The robotic fish has a body and a tail section and moves in water by beating its tail section at preset frequencies; it turns by offsetting the tail beat at an angle to the main body. A real-time visual tracking system uses an overhead camera view to remotely steer the robot into circular trajectories. We use this capability to test the hypothesis that the locomotion of the robotic fish differentially modulates zebrafish behavior. Specifically, we change the speed of the robotic fish by varying the tail-beat frequency of the robotic fish, while keeping the tail beat amplitude constant. To control for the effects of the tail beating and the visual cues from the robot, additional tests are performed in which the robot is beating its tail at a constant frequency in a fixed position in the tank or is completely absent. We expect the following predictions to be met: (i) a free-swimming robot whose design and movement are inspired by a zebrafish will not elicit fear response in zebrafish; (ii) the subjects will change their social interaction in the presence of the robotic fish; and (iii) the speed of the robotic fish will differentially modulate fish collective behavior.

## Materials and Methods

### Ethics statement

The experiments were conducted following the protocols AWOC-2012-101 and AWOC-2013-103 approved by the Animal Welfare Oversight Committee of the Polytechnic Institute of New York University.

### Animals and housing

We used wild-type zebrafish in our experiments. The subjects, approximately 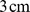 in body length (6–8 months old), were acquired from an online aquarium source (LiveAquaria.com, Rhinelander, Wisconsin, USA). They were maintained in 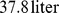 (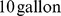) tanks with no more than 20 fish per tank under a 12 hours light/12 hours dark photoperiod [40]. The temperature and pH in the holding tanks were maintained at 27
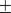
1

C and 7.2, respectively. Fish were fed with commercial flake food (Hagen Corp./Nutrafin max, USA), at approximately 7 pm every day. Experiments were started after a ten day acclimatization period.

### Robotic fish

**Figure 1:**
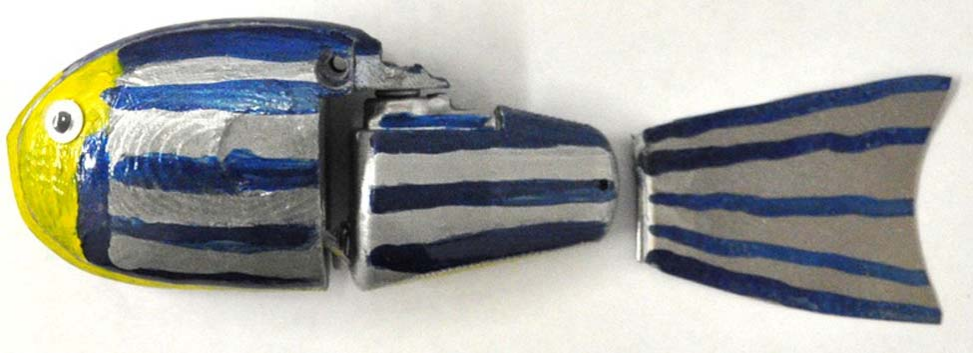
Robotic fish used in our experiments. The color pattern, aspect ratio, and shape of the caudal fin of the mobile robotic fish used in our experiments matched that of a zebrafish.

Following the design in [41], the robotic fish was made of a rigid acrylonitrile butadiene styrene plastic and is divided into a body and tail section (Fig. 1) with a flexible caudal fin. The ratio between the length (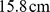), height (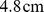), and width (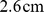) of the robot (aspect ratio) was designed to match that of a fertile zebrafish female. The body section contained a single cell lithium-ion polymer battery, an Arduino Pro mini microcontroller (Sparkfun electronics, USA), and a Nordic nRF2401A transceiver chip (Sparkfun electronics, USA). A Traxxas 2065 servomotor (Traxxas, Plano TX, USA) was inserted into the tail section and was used to actuate the body-tail joint, which, in turn, propelled the robot to move forward. The flexible caudal fin allowed for a sinuous undulation of the tail mimicking carangiform/subcarangiform patterns characteristic of zebrafish [38]. The robot was positively buoyant in water so that it swam just under the surface of the water. The robot was painted bright blue with silver stripes and a yellow head to match zebrafish coloration [42]. Color pattern, aspect ratio, and tail-beat frequency have been shown as relevant factors in the attractiveness of the robotic fish to live zebrafish[12].

The robot was controlled with a remote control unit consisting of an Arduino Duemilanove microcontroller (Sparkfun electronics, USA) and a Nordic nRF2401A transceiver chip (Sparkfun electronics, USA). The microcontroller unit was programmed to receive control parameters namely tail-beat frequency, tail-beat amplitude, and tail-section offset via a universal serial bus (USB). These parameters were then transmitted wirelessly to the robot every 3/5th of a second. In our experiments, we kept the tail-beat amplitude constant at 20 degrees. The heading of the robot was controlled by varying the tail-section offset from a trim value of zero degrees when the robot body was in line with the tail section.

### Experimental setup

**Figure 2:**
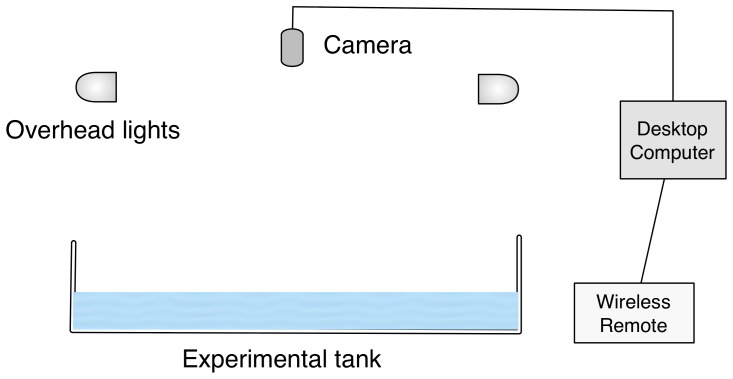
Schematic of the experimental setup. The experimental apparatus consisted of a square shallow tank with overhead ultraviolet lighting and camera for real-time tracking.

The subjects were filmed in a 120 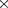 120 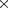 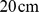 experimental tank supported on an aluminum frame. The water depth was maintained at 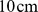 during the experiments. The tank surface was covered with a white contact paper to obtain a high-contrast background for aid in tracking. A Microsoft LifeCam web camera was mounted 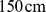 above the water surface to provide a single overhead video feed at a resolution of 640 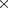 480 pixels through a USB interface. Diffused overhead light from four 25 W fluorescent tubes (All-Glass Aquarium, preheat aquarium lamp, U.K.) was used to illuminate the observation region (Fig. 2). A 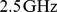 Pentium dual core desktop computer with 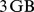 memory ran the real-time multi-target tracking and control algorithm using the camera's input (see Supplementary text for details of tracking and control). The setup was isolated during the experiment with dark curtains. Experimental data consisting of image frames and trajectories, was backed up every night and a duplicate copy maintained for analysis.

### Experimental procedure

The experimental conditions consisted of the robot swimming in the experimental tank with three zebrafish (Fig. S1 in File S1). Four conditions were tested to evaluate the hypothesis that the locomotion of a robotic fish differentially modulates the behavior of zebrafish groups. These conditions comprised a robot not beating its tail in a static position, and then beating its tail at 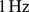, 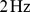, and 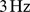 as it swam in circular trajectories of constant radius. For brevity, these conditions are referred to as 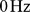, 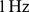, 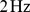, and 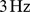. These tail-beat frequencies correspond to robot speeds of approximately 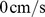, 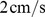, 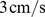, and 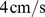, respectively, and were selected to explore variations in the reference condition corresponding to 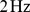, which was considered in the preference tests described in [12]–[14], [16]. To compensate for the effect of noise, the servo motor was kept on during the 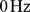 condition although the tail of the robot was not moving.

**Figure 3:**
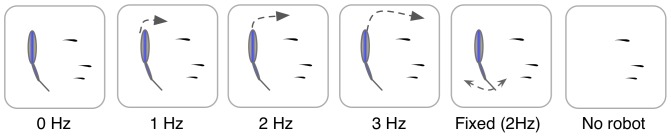
A summary of the experimental conditions. We considered tail-beat frequencies of 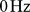, 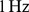, 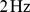, and 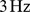 corresponding to swimming speeds of 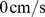, 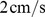, 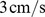, and 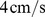. To control for the tail-beating movement and the presence of the robot, we conducted tests with the robot anchored to preset locations in the tank and without the robot.

To control for the effects of the tail-beat and for the visual cues associated with the presence of the robot in the tank, we conducted two additional experimental conditions. In one condition, called Fixed, the robot was held in a place within the tank at different positions with its tail beating at 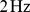 and in the other condition, called No robot, the fish were observed without the robot in the tank. The radius of the circular trajectory, whenever the robot was swimming, was set at a constant value of 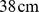 (Fig. S2 in File S1). A total of 144 subjects, distributed over 48 trials with three naive subjects in each trial, were used to conduct the experiments (Fig. 3).

In each trial, fish in groups of three were transferred into the experimental tank with a hand net. The tracking and control system was initiated immediately after. The tracking system automatically computed the background and intensity threshold. The intensity threshold was lowered from a high value in successive frames until three distinct targets were obtained consistently. The robot communication and trim settings were tested next by beating the tail about the center, followed by 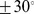 offset. After testing, the robot was positioned at a predetermined starting point located near the tank corner. A habituation time of ten minutes with the robot set stationary was given to avoid novelty effects [13], [18], [43], [44]. During this time, the servomotor was turned on with the tail-beat amplitude set to zero to create a uniform acoustic background. The start signal was transmitted to the robot at the end of the habituation time followed by a five minute experimental session. For experiments in which the robot was stationary during the trial time (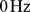 and Fixed), the robot was anchored from the bottom to a transparent plexiglass sheet at equally spaced locations around the 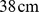 circular trajectory.

### Data analysis

The experiments were recorded at five frames per second for five minutes each, resulting in a total of 1500 frames per trial. Trajectory data was verified and repaired if needed for each individual trial using a custom MATLAB script.

The degree of cohesion of zebrafish groups was described in terms of the average nearest-neighbor distance (ANND) [45], [46]. Given the two-dimensional position 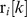 of the 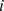-th fish at frame 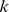, the ANND during that frame is [46]

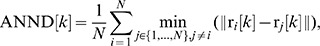
(1)where 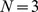 is the total number of fish and 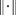 denotes the standard Euclidean norm. Group coordination was captured through the polarization (Pol) that measures the degree of alignment between the directions of motion of the fish [47]. Given the two-dimensional velocity 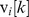 of the 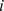-th fish at frame 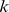, the polarization at that frame is
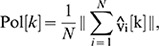
(2)where 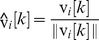 is the direction of motion. The group speed given by 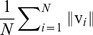 was computed to describe average speed of the subjects. Group interaction with the robotic fish was quantified in terms of three values, namely, the average distance to the robot, the minimum distance to the robot, and the relative group speed. Minimum distance to the robot was computed by comparing the individual fish distances to the robot center on the image, and relative group speed is defined as the difference between group speed and the speed of the robot.

To elucidate the behavior of the subjects, we scored the time spent freezing during each trial. This value was computed automatically from the trajectory data. In particular a fish was considered freezing during a frame if it spent two continuous seconds within a ball of radius of 2 cm [14]. Freezing was recorded if at least one fish satisfied this condition.

To evaluate the effect of robot locomotion, a one-way analysis of variance (ANOVA) with the ANND, Pol, average speed, average distance to the robot, minimum distance to the robot, and time spent freezing, each as a dependent variable, and the robot speed as the independent variable was used. Tukey HSD post-hoc tests were performed to compare these quantities between pairs of robot speeds if a significant effect was found. Statistical power for the given sample size of 32 (4 conditions 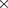
8 experimental groups each) was confirmed (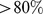) using average nearest neighbor distance and speed as the measurement variables. In particular, corresponding values of shoaling and schooling from [32] were used to compute the effect size (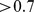) with the expectation that over the full range of robot speeds the values of ANND and group speed will change one body length and 3 cm/s respectively. To evaluate the effect of time, a two-way ANOVA with the same dependent variables, but time of observation in minutes and robot speed as the independent variables (eight replicates for each minute of the five minute experimental session), was performed. One-way ANOVA was used to compare control conditions with select test conditions. Significance level for all tests was set at p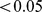. Statistics were computed using MATLAB (R2011a, Mathworks) and power analysis was performed using G*Power 3.1 [48].

## Results

### Group cohesion

**Figure 4:**
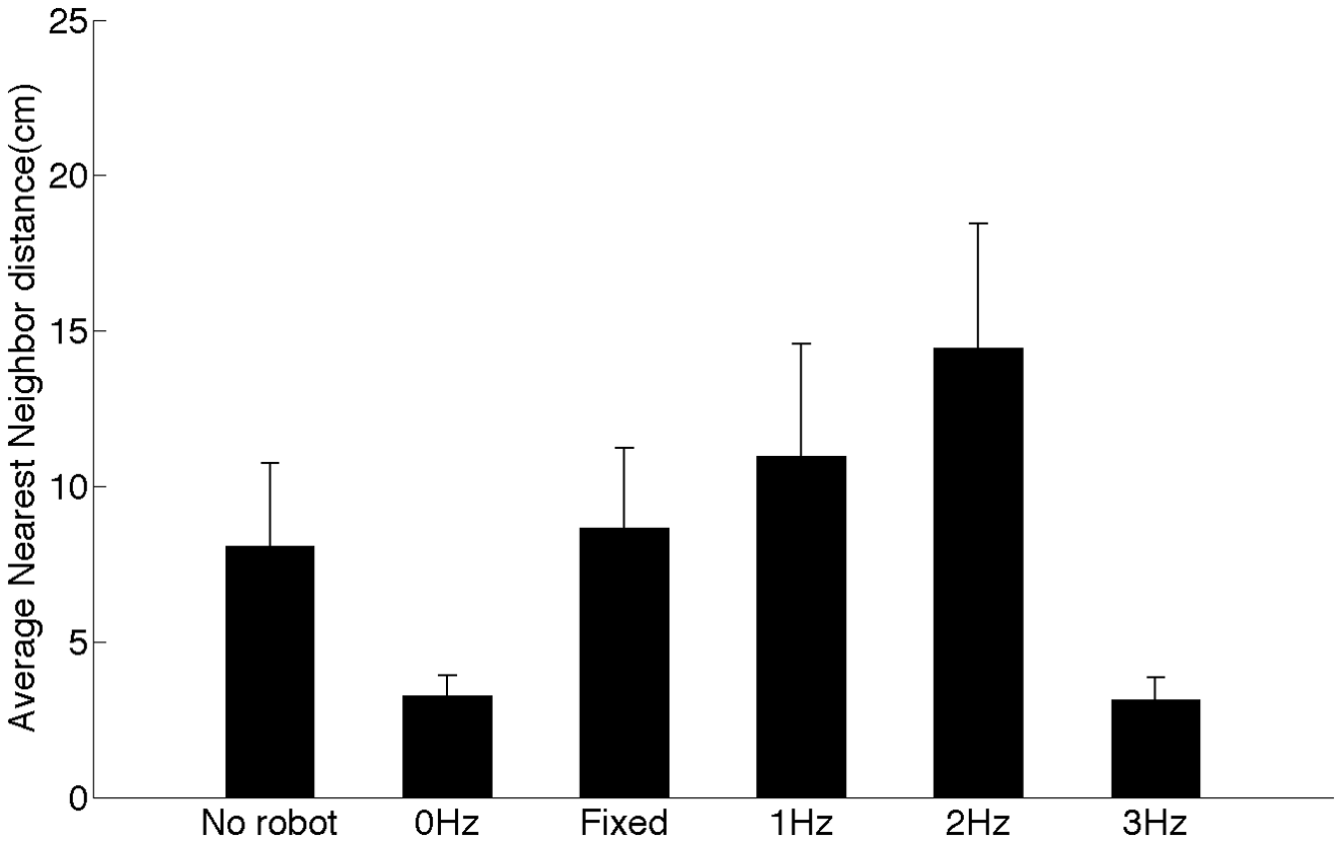
Group cohesion changed significantly with robot speed. Average nearest-neighbor distance between zebrafish as the robot moved at increasing speeds of 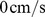, 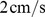, 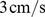 and 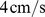 corresponding to a tail-beat frequency of 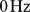, 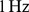, 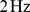, and 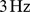 respectively. Control conditions (No robot and Fixed) are shown for reference. Error bars represent 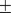 standard error mean.

A comparison of ANND values across test conditions (Fig. 4) indicates a significant difference with change in robot speed (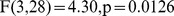). The value of ANND initially rises with the robot speed and then drops as the robot speed is increased further. Post-hoc analysis shows that the maximum (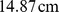) and minimum (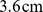) values of ANND are attained at robot speeds of 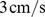 and 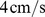 respectively. Individual comparisons show that the maximum value of ANND is significantly different from those attained when the robot is stationary and when the robot is swimming at 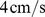. We do not observe a significant effect of the presence of the robot (No robot and 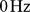, p = 0.099) and effect of tail-beat movement only (Fixed and 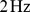, p = 0.243) on group cohesion.

### Group coordination

**Figure 5:**
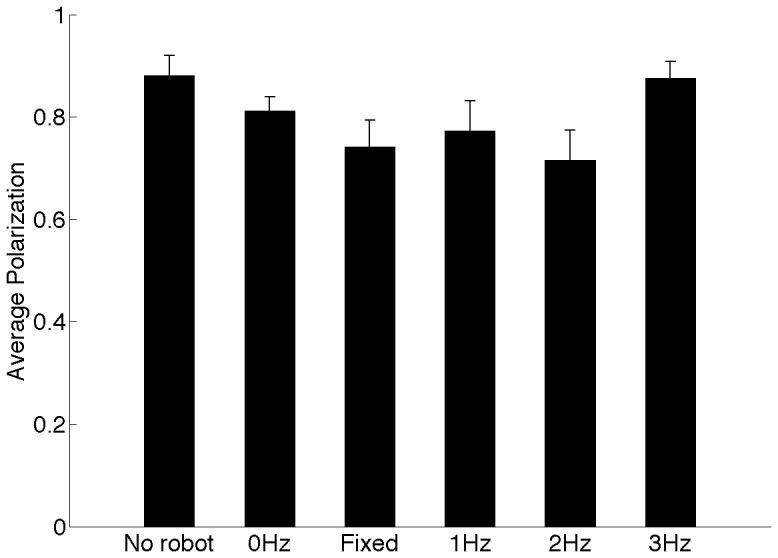
Group coordination was always high. The polarization of zebrafish was not significantly different between conditions with the robot moving at varying speeds (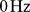, 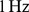, 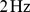, and 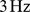). Control conditions (No robot and Fixed) are shown for reference. Error bars represent 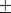 standard error mean.

Figure 5 compares the polarization of the groups as a function of the robot speed. Therein, we observe an initial drop in the polarization as the robot speed increases from 0–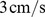 followed by an increase at the robot speed of 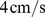, however the effect of the robot speed on the polarization fails to reach statistical significance (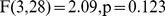). We find that the presence of the robot (No robot and 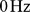, p = 0.172) and the effect of tail-beat movement only (Fixed and 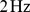, p = 0.740) do not change the coordination in zebrafish. Polarization distributions are bimodal (Fig. S7 in File S1) with the fish being highly polarized for the majority of the time across conditions.

### Group speed

The group speed of the fish is not significantly affected by the robot speed (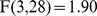, 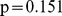). While the tail-beat movement of the robot does not produce a significant effect on group speed (Fixed and 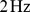, p = 0.375), the presence of the robot produces a significant change in the speed of the subjects (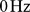 and No robot, p = 0.0001). The highest value of group speed of 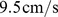 is observed in the absence of the robot (Fig. S3).

### Fish-robot interactions

**Figure 6:**
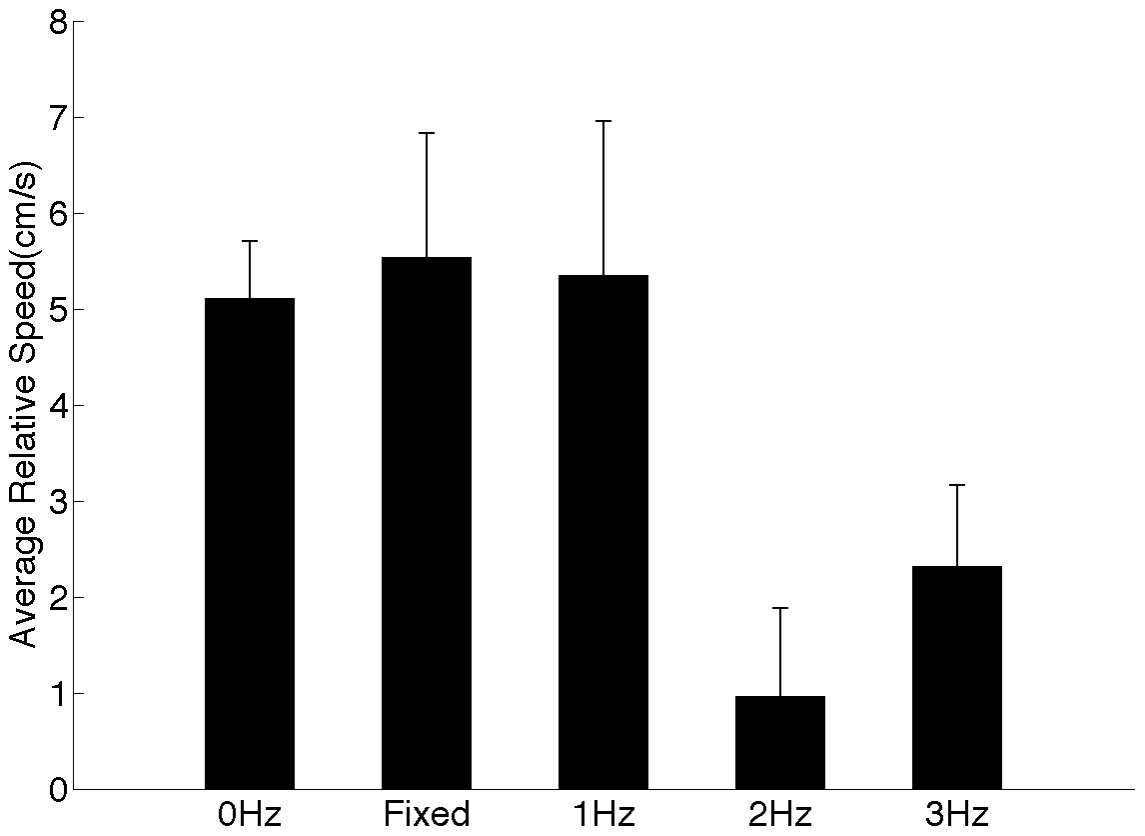
Group speed relative to the robot varied with robot speed. The relative speed of zebrafish was significantly different between conditions with the robot moving at varying speeds (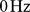, 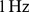, 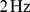, and 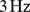). Control conditions (No robot and Fixed) are shown for reference. Error bars represent 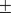 standard error mean.

The average (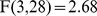, 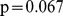) and the minimum distance of the zebrafish to the robot (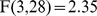, 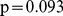) appear to be related to the robot speed, however none of these dependencies reach statistical significance (Figs. S5 and S6 in File S1). The average distance of the fish to the robot is more than 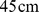. The largest difference of 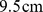 between the average and the minimum distance to the robot is observed in the 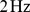 condition. Relative group speed (Fig. 6) changes significantly with the robot speed (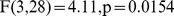), with post-hoc analysis showing that the least difference of 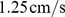 (at robot speed at 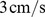) is significantly different from values when the robot is stationary and swimming at 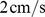.

### Group behavior

Between the test conditions, the speed of the robot does not alter the time spent freezing (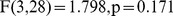). Similarly, neither the presence of the robot (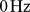 and No robot, p = 0.091), nor the effect of tail-beat movement only (Fixed and 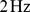, p = 0.689) significantly changes the time spent freezing by the zebrafish, although the mere presence appears to increase this time by a small percentage (Fig. S4 in File S1).

### Time-effect

The effect of time is not significant in robot speed on cohesion (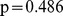), polarization (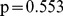) and speed (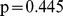), distance to the robot (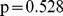), minimum distance to the robot (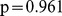), and time spent freezing (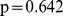) with no significant interaction between robot speed and time (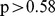 for all values).

## Discussion

The results of this study confirm that the robotic fish is not perceived as a conspecific by the zebrafish [13], whereby fish tend to maintain a considerably smaller distance between themselves than with the robot. At the same time, we discount the effects of novelty-induced fear to explain high cohesion in any of the conditions because of the ten minute habituation time. This value is almost twice the six minute habituation time that has been shown to significantly increase exploratory behavior and decrease instances of freezing in zebrafish [43], even in the presence of a predator model [18]. This inference is also supported on the basis of prior studies with the same robot, where a consistent spatial preference for the robot was found with similar habituation times [12], [13].

The results indicate that 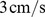, obtained with a tail-beat frequency of 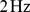, is a critical speed for the robotic fish, with increases above or below this threshold differentially affecting zebrafish collective response. In particular, we observe a significant decrease in the group cohesion and a continued decrease in the polarization of the zebrafish shoals as the robot increases its speed from 0 to 3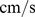. The cohesion suddenly increases as the robotic fish speed is increased to 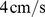 and is accompanied by a similar, though not significant, increase in polarization. Furthermore, at this tail beat frequency, we also find that fish vary their speed to match the robot speed, while exhibiting the maximum disparity between the average and the minimum distance from it. The latter evidence indicates that fish tend to leave the shoal to interact with the robotic fish when it beats the tail at 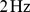. The variation in fish response as a function of robot speed is unlikely to be related to sensory cues generated by the body movement only, since the fish do not behave differently if the robot is fixed and beats its tail at 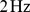. Therefore, we propose that visual cues associated with the motion of the robot at 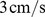 are relevant factors in shaping the interaction between the live subjects and the robotic fish.

It is possible that the robot is perceived as a predator in conditions where it is stationary or moving slowly. This argument is supported by findings in [2], which indicate that zebrafish reduce their speed when confronted with a computer animated image of a predator. The hypothesis that the robot is perceived as a predator is also favored by the increase of fish cohesion as the robot is present in the tank. Indeed, an increase in cohesion is indicative of response to predatory threats [45], [49], alarm substances [50], and novelty-induced fear [51]. However, this change is not accompanied by an increased polarization, which is also associated with high threat perception in fish schools [32], [52]; in fact, polarization decreases with the introduction of the robot. The bimodal distributions of polarization show that the fish spend time either shoaling or schooling, suggesting that the decrease in polarization is due to an increased shoaling tendency of the fish. A second result that does not support the possibility that the robot is perceived as a predator is the absence of a time effect in the group interaction, quantified in terms of minimum and average distance to the robot. This is in contrast with experimental studies that demonstrate the increased avoidance of a predator with time in the form of increased distance from it [44]. Although this independence over time can also be explained by a ceiling effect, zebrafish do not maximize their distance to the robot, ranging between 40–60 cm, in an environment that allows for maintaining distances as large as 90–100 cm. Finally, the freezing response of the subjects does not increase in the presence of the robot, as we would expect if the robot was perceived as a predator [44], [53].

The presence of results both in favor and against the robot being perceived as a predator by the zebrafish posits the need for further studies on zebrafish perception of the robot motion. Although we did not measure the tail-beat frequency and amplitude of zebrafish in our experiments, the robot was designed in [12], [41] to mimic the carangiform/subcarangiform swimming motion of zebrafish [54]. In particular, the tail-section and passive caudal fin were approximately half the total body length of the robot. Despite offering a considerably less tail-beat frequency and speed than live zebrafish [55], the robot's locomotion is unlike a sympatric predator that would typically consist of short bursts of motion accompanied with long durations of stationary behavior [44]. Perhaps the visual cues associated with this slow, yet persistent, motion of the robot reduce the possibility of it being perceived as a predator.

This work shows that both body and spatial movements of the robotic fish differentially modulate zebrafish behavior. Specifically, by controlling for spatial movement and body movement separately we are able to highlight a combination where the fish tend to explore the unconstrained free-swimming environment. The ability to actively interact with fish in a large unconstrained environment is expected to improve our understanding of shoaling behavior [31], [38], [42]. The system described here is scalable with respect to both robots and animals and can track their behavior in real time.

Future work will be driven in several directions, including the dependence of zebrafish shoal size on its response [56] to the robotic fish, and conversely, the dependence of the size and configuration of a shoal of robotic fish on zebrafish response.

## Supporting Information

File S1 Supporting information text detailing the multi-target tracking method, robot control design, and synopsis of experimental data on fish response.
(PDF)
