# Supplementary material for: Collective Response of Zebrafish Shoals to a Free-Swimming Robotic Fish
Source: PLoS One. 2013 Oct 16;8(10):e76123. doi: 10.1371/journal.pone.0076123 (PMC3797741; doi:10.1371/journal.pone.0076123)
Supplement: File S1 — Supporting information text detailing the multi-target tracking method, robot control design, and synopsis of experimental data on fish response. (PDF) [file pone.0076123.s001.pdf]

Supporting information: Collective response of zebrafish shoals to a free-swimming robotic fish

Multi-target tracking

The control input to the robotic fish is computed using a real-time two-dimensional multi-target tracking algorithm developed in MATLAB. The tracking algorithm takes as input 640×480 pixel raw image frames at five frames per second from an overhead camera and outputs the position and velocity estimates for each target in the tank.

Our goal is to estimate the position, velocity, and size of the fish and the robot as they move within the experimental tank. We use these values to describe the instantaneous state of the target i at a frame k given by $\boldsymbol{X}_i[k] = \begin{bmatrix} \boldsymbol{r}_i & \boldsymbol{v}_i & A_i & 1 \end{bmatrix}^{\mathrm{T}}$, where $\boldsymbol{r}_i \in \mathbb{R}^2$ is the two-dimensional position, $\boldsymbol{v}_i \in \mathbb{R}^2$ is the two-dimensional velocity, and A_i is the area in pixels. The state vector is augmented with 1 so that the mapping from pixel to cm (measurement model) is linear. The measurement of the target i, $\boldsymbol{Z}_i[k] \in \mathbb{R}^3$ consists of two-dimensional pixel measurement of the center of the target and the area in pixels.

To obtain the position measurements in a frame, we first isolate the targets on the image plane by subtracting a running background computed from raw images [1]. The resulting foreground consists of pixel blobs whose center is recorded as two-dimensional position measurement. The robot is identified on the basis of the blob size on the image (> 200 pixels).

We implement a Bayesian framework to recursively predict the state estimate at a future time-step using a motion model, F, and update the same at the current time-step using a measurement model, H. The state estimate and measurement for target i at a future frame $k+1$ is related to the current estimate at k according to

$$\begin{aligned} \boldsymbol{X}_i[k+1] &= F(\boldsymbol{X}_i[k], \boldsymbol{\omega}) \\ \boldsymbol{Z}_i[k+1] &= H(\boldsymbol{X}_i[k+1], \boldsymbol{\eta}), \end{aligned} \tag{1}$$

where $\boldsymbol{\omega} \in \mathbb{R}^6$ is the disturbance, and $\boldsymbol{\eta} \in \mathbb{R}^3$ is the measurement noise. If (1) is linear and $\boldsymbol{\omega}$ and $\boldsymbol{\eta}$ are Gaussian white, the state $\boldsymbol{X}_i$ and measurement $\boldsymbol{Z}_i$ are also Gaussian and (1) can be written in matrix form. With linear models and Gaussian representation, given a measurement $Z_i[k]$, a Kalman filter estimates the state $\boldsymbol{X}_i[k]$ optimally in the sense of minimum mean square error [2–4].

Assuming $\boldsymbol{\omega}$ and $\boldsymbol{\eta}$ to be Gaussian, we model $F \in \mathbb{R}^{6\times 6}$ as a constant velocity motion model [3] of the form

$$F = \begin{bmatrix} 1 & 0 & \Delta t & 0 & 0 & 0 \\ 0 & 1 & 0 & \Delta t & 0 & 0 \\ 0 & 0 & 1 & 0 & 0 & 0 \\ 0 & 0 & 0 & 1 & 0 & 0 \\ 0 & 0 & 0 & 0 & 1 & 0 \\ 0 & 0 & 0 & 0 & 0 & 1 \end{bmatrix}, \tag{2}$$

where $\Delta t = 0.2$ (in seconds) is the time difference between successive frames. Given a pixel to cm ratio p computed using select points on the image known distance apart and the center of the observation region $\left[c_1, c_2\right]^{\mathrm{T}}$, the measurement model H is

$$H = \begin{bmatrix} p & 0 & 0 & 0 & 0 & c_1 \\ 0 & p & 0 & 0 & 0 & c_2 \\ 0 & 0 & 0 & 0 & 1 & 0 \end{bmatrix}. \tag{3}$$

The covariance for $\boldsymbol{\omega}$ and $\boldsymbol{\eta}$ are diagonal matrices $\mathrm{cov}(\boldsymbol{\omega}) = \mathrm{diag}\{1, 1, 10, 10, 2, 0\}$ (in cm^2, cm^2, cm^2/s^2 cm^2/s^2, pixels^2) and $\mathrm{cov}(\boldsymbol{\eta}) = \mathrm{diag}\{1, 1, 16\}$ (in pixels^2, pixels^2, pixels^2). The output at each step of the Kalman filter is a the mean and covariance of the state.

For tracking multiple targets, the correct measurement must be associated to each target before a measurement update is performed. We use a global nearest-neighbor algorithm that minimizes the combined distance between measurements and target estimates to match measurements to targets [5, 6]. Targets are automatically initialized with unassociated measurements and sustained until no measurement is found within a confidence region defined by a two-dimensional Chi-square distribution [3, 7]. Upon an occlusion when two blobs merge into one on the foreground, one of the tracks is automatically terminated and re-initialized after the occlusion. The robotic fish is rarely lost during the five-minute trial period, although the fish, due to their small size (2-3 pixels on the image) and water surface ripples may get lost and recovered more frequently. Background clutter and noise is reduced through careful use of lighting and a single-color surface. The estimates along with the error covariance and control inputs are written to a data file for subsequent analysis. Figure S1 shows robot and fish trajectories during the last minute in a five-minute trial.

Robot control

The estimates of robot position $\boldsymbol{r}_R[k]$ and velocity $\boldsymbol{v}_R[k]$ are used to calculate the control input $u[k]$. The control signal is sent every 3/5-th of a second. The robot follows a set of sixteen equally spaced waypoints $\boldsymbol{w}_s, s = 1, \ldots, 16$ clockwise on a 40 cm circle centered in the tank. At each frame k, the desired direction of movement of the robot is computed

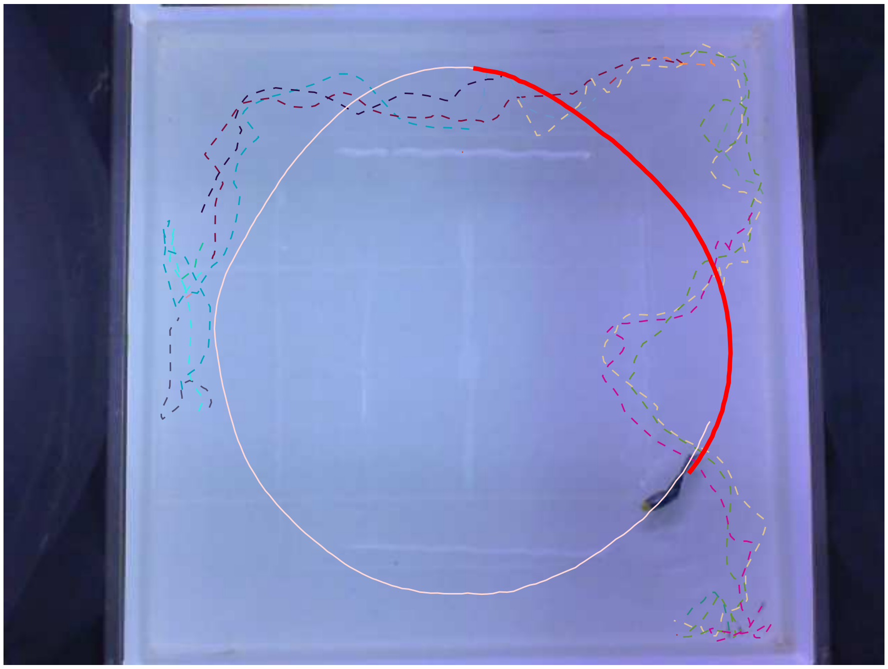

Figure S1. Tracks of zebrafish (dashed) with the robot(solid) from the last minute in a five minute experiment.

as

$$\hat{\boldsymbol{v}}_R^d[k] = \frac{\boldsymbol{w}_s[k] - \boldsymbol{r}_R[k]}{\|\boldsymbol{w}_s[k] - \boldsymbol{r}_R[k]\|}, \tag{4}$$

where $\boldsymbol{w}_s[k]$ is the waypoint-to-reach at the current time-step. The value of waypoint-to-reach is updated at frame k' when the robot reaches within a threshold distance of the current waypoint-to-reach. The control input is a function of the error $e = \sin(\theta)$, where $\theta = \arg(\hat{\boldsymbol{v}}_R[k] - \boldsymbol{v}_R^d[k)$ is the angle between the robot direction of motion and the desired direction of motion. The control input is computed in a PID loop as

$$u[k] = K_p e[k] + K_i \sum_{l=k'}^{k} e[l]\Delta t + K_d \frac{\Delta e[k]}{\Delta t}, \tag{5}$$

where K_p, K_i, and K_d is the proportional, integral and derivative control gains and $\Delta e[k] = e[k] - e[k-1]$. The control gains are tuned to obtain the minimum error in test trials performed over five minutes. Figure S2 compares the robot trajectory to the

waypoints on the tank region, and shows the corresponding error through time. For additional details on the tracking and control algorithm and its implementation refer to [8].

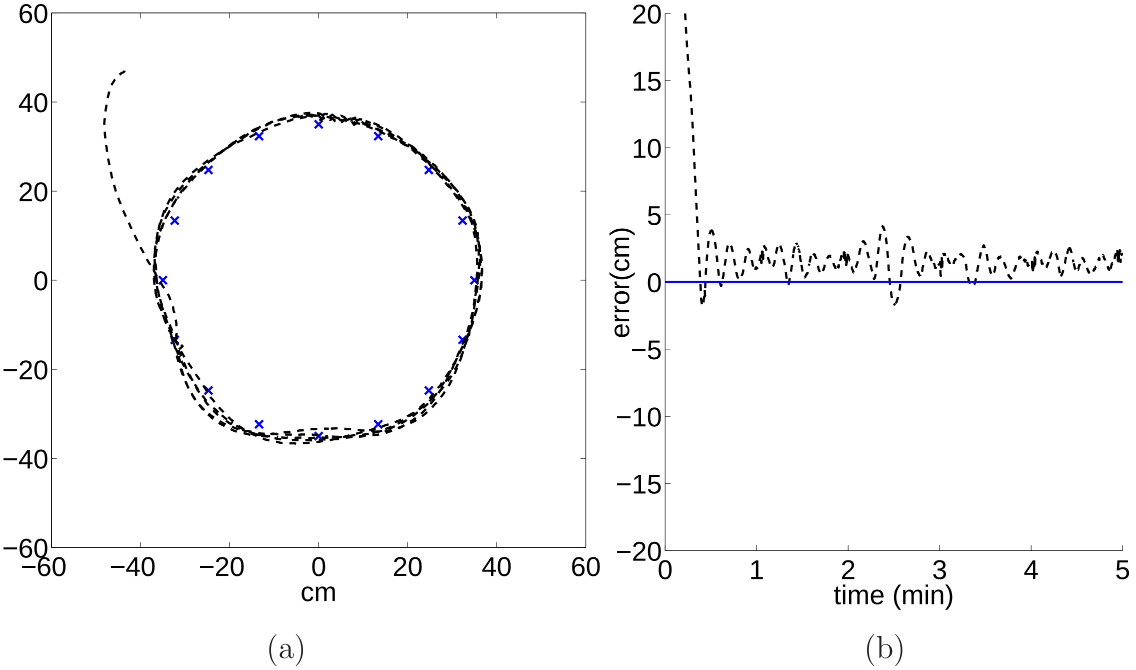

Figure S2. Robot trajectory with reference to waypoints (a) and the error (b).

Synopsis of experimental data on fish response

In this section, we compare values of group response, behavior, and interaction with the robot between all experimental conditions performed as part of this study. In addition to the main text, we show the values of group speed (Fig. S3), for the group response; average percentage time spent freezing (Fig. S4) for the group behavior; and distance to robot (Fig. S5), and minimum distance to robot (Fig. S6) for the interaction with the robot. For experiments where group response and behavior are measured, the values are shown for conditions, No robot, Fixed, 0 Hz, 1 Hz, 2 Hz, and 3 Hz. For experiments where group interaction with the robot is measured, values are shown for all conditions except where the robot is absent.

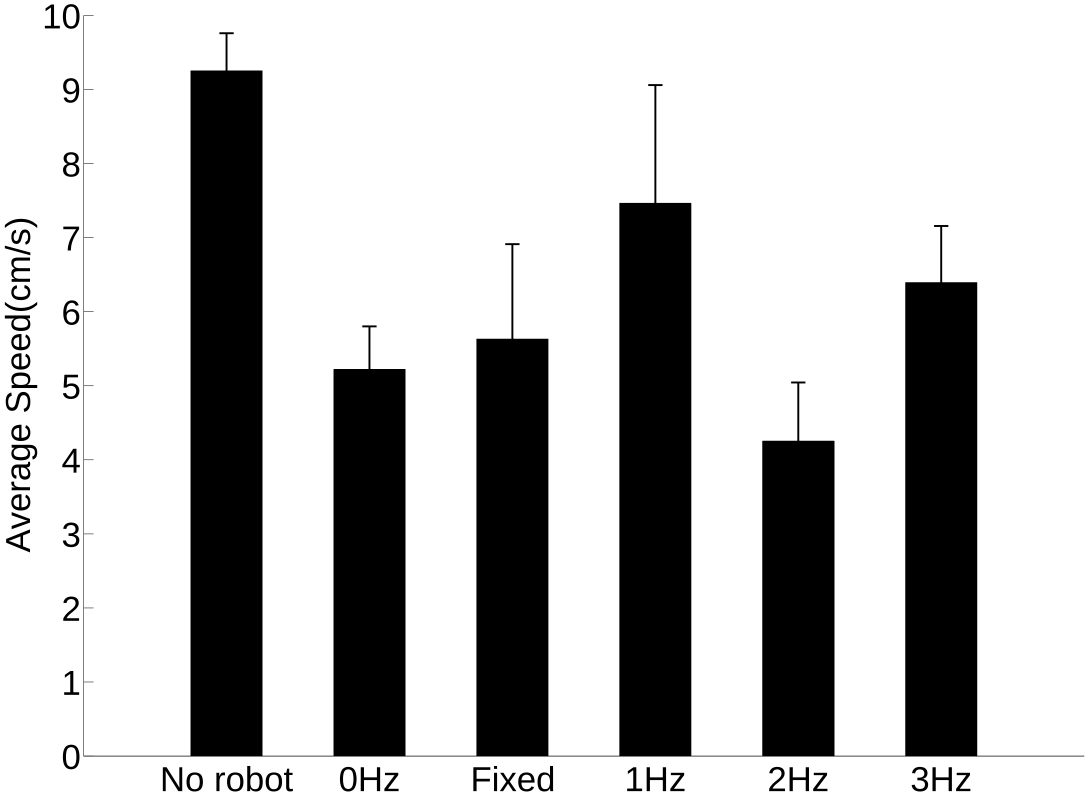

Figure S3. Average values of speed across all conditions. The highest group speed was when the robot was not present, and lowest when the robot was swimming with a tail-beat frequency of 2 Hz. Error bars represent ± standard error mean.

References

1. Butail S, Paley DA (2011) Three-dimensional reconstruction of the fast-start swimming kinematics of densely schooling fish. Journal of the Royal Society Interface 9: 77–88.

2. Brown RG (1983) Introduction to random signals and applied Kalman filtering. New York, NY, USA: John Wiley & Sons.

3. Bar-Shalom Y (1987) Tracking and data association. San Diego, CA, USA: Academic Press Professional, Inc.

4. Yilmaz A, Javed O, Shah M (2006) Object tracking: A survey. ACM Computing Surveys (CSUR) 38: 1–45.

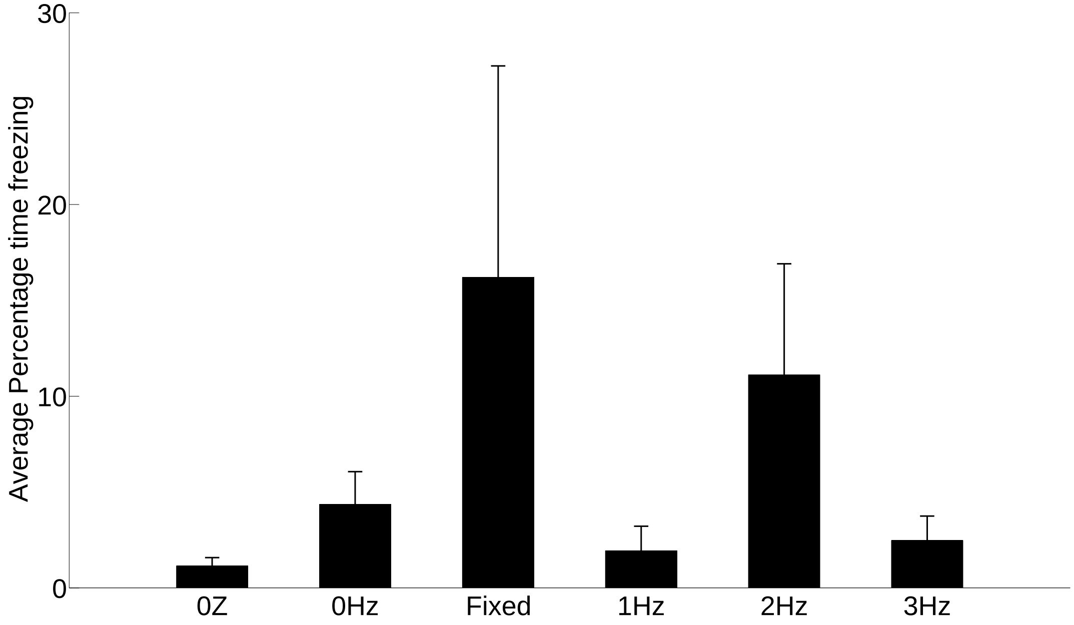

Figure S4. Average values of percentage time freezing across all conditions. Error bars represent ± standard error mean. The lowest percentage time was when no robot was present and the highest when the robot was swimming with a tail-beat frequency of 2 Hz, although this value also had the largest error bar.

5. Kuhn HW (1955) The Hungarian method for the assignment problem. Naval Research Logistic Quarterly 2: 83–97.

6. Munkres J (1957) Algorithms for the Assignment and Transportation Problems. Journal of Society of Industrial and Applied Mathematics 5: 32–38.

7. Reid D (1979) An algorithm for tracking multiple targets. IEEE Transactions on Automatic Control 24: 843–854.

8. Butail S, Bartolini T, Porfiri M (2013) Collective response of zebrafish to a mobile robotic fish (accepted). In: Proceedings of the ASME Dynamic Systems and Control Conference.

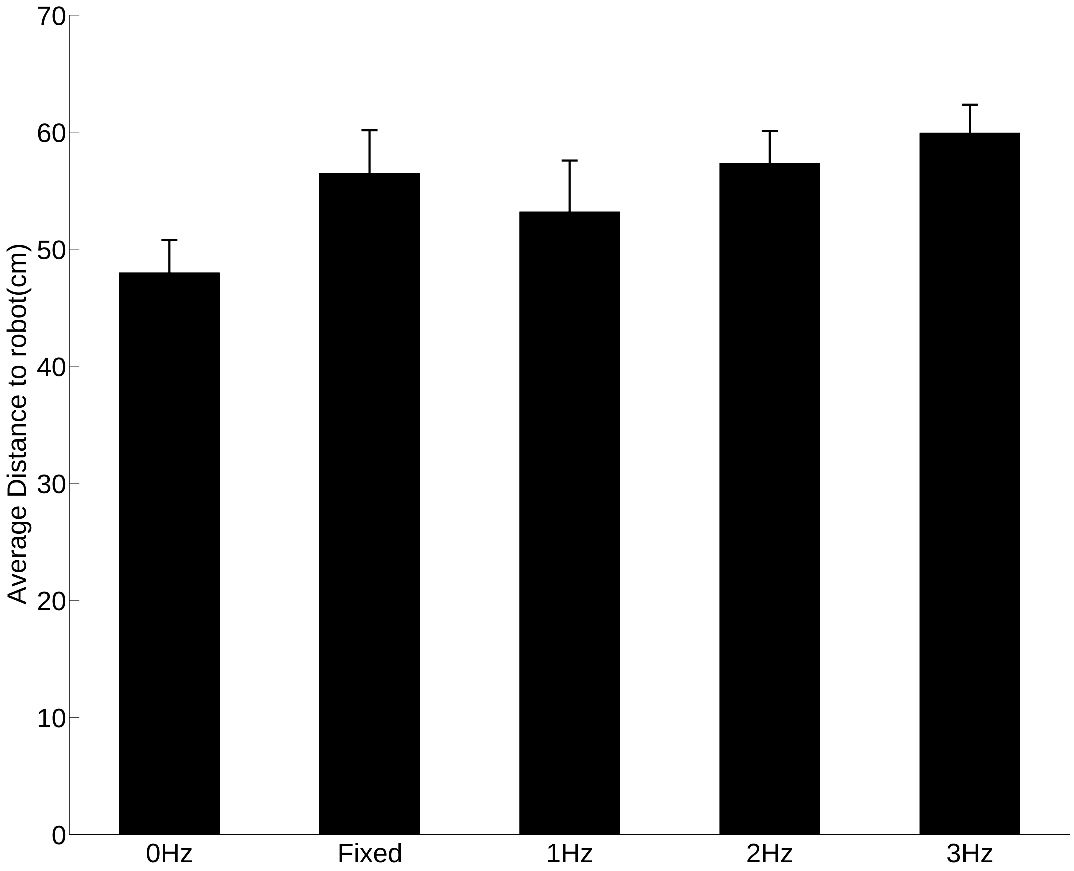

Figure S5. Average values of distance to robot across all conditions. The fish were on average closest to the robot when it was not moving at all, and furthest when the robot was swimming with a tail-beat frequency of 3 Hz. Error bars represent ± standard error mean.

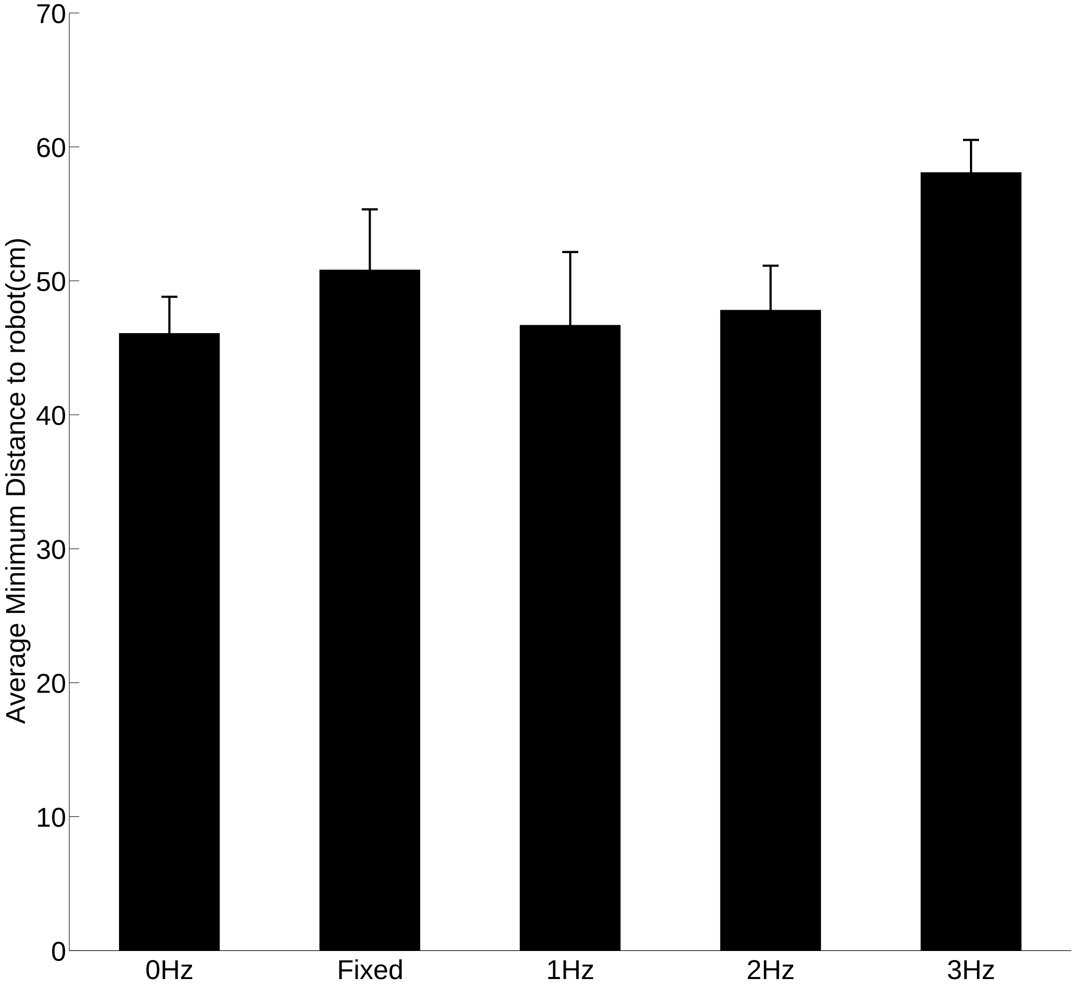

Figure S6. Average values of minimum distance to robot across all conditions. At any time, the minimum distance was computed by comparing individual distances of the fish to the robot. The highest and the lowest values were at 3 Hz and 0 Hz respectively. Error bars represent ± standard error mean.

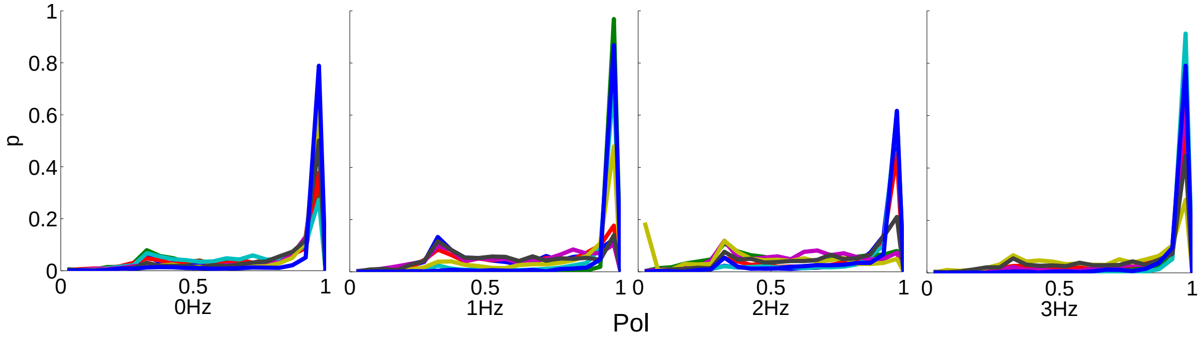

Figure S7. The polarization distributions had two distinct peaks at low and high values. Polarization distributions for each trial are shown in different colors.
